# Metabolic effects of newly synthesized phosphodiesterase-3 inhibitor 6-[4-(4-methylpiperidin-1-yl)-4-oxobutoxy]-4-methylquinolin-2(1H)-one on rat adipocytes

**DOI:** 10.1186/s40199-015-0100-2

**Published:** 2015-02-21

**Authors:** Bagher Alinejad, Reza Shafiee-Nick, Hamid Sadeghian, Ahmad Ghorbani

**Affiliations:** Department of Pharmacology, School of Medicine, Mashhad University of Medical Sciences, Mashhad, Iran; Pharmacological Research Center of Medicinal Plants, School of Medicine, Mashhad University of Medical Sciences, Mashhad, Iran; Department of laboratory Sciences, School of Paramedical Sciences, Mashhad University of Medical Sciences, Mashhad, Iran

**Keywords:** Adipogenesis, Amrinone, Cilostamide, Lipolysis, Phosphodiesterases

## Abstract

**Background:**

Clinical use of selective PDE3 inhibitors as cardiotonic agents is limited because of their chronotropic and lipolytic side effects. In our previous work, we synthesized a new PDE3 inhibitor named MC2 (6-[4-(4-methylpiperidin-1-yl)-4-oxobutoxy]-4-methylquinolin-2(1H)-one) which produced a high positive inotropic action with a negative chronotropic effect. This work was done to evaluate the effects of MC2 on adipocytes and compare its effects with those of amrinone and cilostamide.

**Methods:**

Preadipocytes were isolated from rat adipose tissue and differentiated to adipocyte in the presence of cilostamide, amrinone or MC2. Lipolysis and adipogenesis was evaluated by measuring glycerol level and Oil Red O staining, respectively. Adipocyte proliferation and apoptosis were determined with MTT assay and Annexin V/PI staining, respectively.

**Results:**

Differentiation to adipocyte was induced by amrinone but not by cilostamide or MC2. Basal and isoproterenol-stimulated lipolysis significantly increased by cilostamide (p < 0.05). Similarly, amrinone enhanced the stimulated lipolysis (p < 0.01). On the other hand, MC2 significantly decreased both adipogenesis (p < 0.05) and stimulated lipolysis (p < 0.001). Also, incubation of differentiated adipocytes with MC2 caused the loss of cell viability, which was associated with the elevation in apoptotic rate (p < 0.05).

**Conclusion:**

Our data indicate that selective PDE3 inhibitors produce differential effects on adipogenesis and lipolysis. MC2 has proapoptotic and antilipolytic effects on adipocytes and does not stimulate adipogenesis. Therefore, in comparison with the clinically available selective PDE3 inhibitors, MC2 has lowest metabolic side effects and might be a good candidate for treatment of congestive heart failure.

## Introduction

Cyclic nucleotide phosphodiesterases (PDEs) control the level of intracellular cAMP and cGMP, and hence play important roles in cellular signaling pathways. The PDEs are grouped into 11 families which differ in their physiochemical properties, substrate specificities, tissue distributions, and regulatory mechanisms [1]. Among them PDE3 has high expression in heart, airway, liver, pancreas, and adipose tissue and involves in the regulation of cardiovascular functions, insulin secretion, and lipid metabolism [2-5]. Selective PDE3 inhibitors have vasodilatory, antithrombotic, antiproliferative, bronchodilatory, anti-inflammatory, and positive inotropic effects [6-8]. A number of PDE3 inhibitors including cilostamide, cilostazol, milirinone, and amrinone were developed to treat patients with heart failure. However, chronic treatment with these agents was associated with some life-threatening side effects, arrhythmia in particular [7-10]. Therefore, designing new PDE3 inhibitors with desired pharmacological properties and lesser side effects is an attractive subject.

In the previous works we synthesized novel analogs of cilostamide as selective PDE3 inhibitors and evaluated their cardiac and metabolic properties. One of the test compounds, named MC2 (6-[4-(4-methylpiperidin-1-yl)-4-oxobutoxy]-4-methylquinolin-2(1H)-one), produced a potent inotropic action without unwanted effect on basal contraction rate [11-14]. Therefore, it may be a candidate for use as a PDE inhibitor in patients with cardiovascular diseases.

However, in adipose tissue, PDE3 inhibitors increase intracellular cAMP level and thereby increases lipolysis in adipocytes and enhance adipogenesis in preadipocytes [15,16]. Dysregulation of lipid metabolism in adipose tissue may cause deleterious consequences in some pathological conditions such as diabetes mellitus, insulin resistance, fatty liver, and obesity [17-19]. Therefore, as a selective PDE3 inhibitor, MC2 is expected to produce some effects on lipid metabolism which could be a potential risk factor for clinical adverse effect.

Therefore, in the present work, we evaluated the effects of MC2 on adipose tissue functions including adipogenesis and lipolysis, and also on adipocyte viability and apoptosis. Furthermore, the effects of MC2 were compared with the effects of amrinone and cilostamide on adipose tissue.

## Materials and method

### Chemicals and reagents

Isoproterenol, free glycerol reagent, 4-(2-hydroxyethyl) piperazine-1-ethanesulfonic acid sodium salt (HEPES), 4, 5-Dimethylthiazol-2-yl, 2, 5-diphenyl tetrazolium (MTT), fatty acid-free bovine serum albumin (BSA), and collagenase were purchased from Sigma (USA). Dimethyl sulfoxide (DMSO) and 3-isobutyl-1-methylxanthine (IBMX) were provided from Fluka (Buchs, Switzerland). Dulbecco’s modified eagle’s medium (DMEM), fetal bovine serum (FBS), penicillin/streptomycin, and Annexin V/PI apoptosis kit were obtained from Invitrogen (USA). Indomethacin, dexamethasone, and insulin were kindly provided by EXIR Company (Iran).

**Figure 1:**
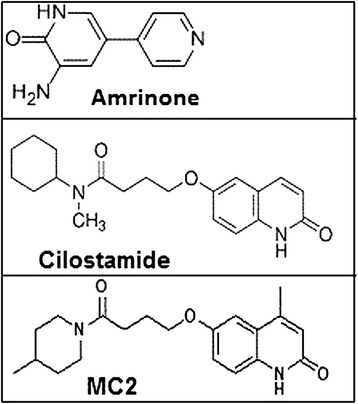
**Structure of phosphodiestrase-3 inhibitors amrinone (IC**_**50**_ **~ 50 μM), cilostamide (IC**_**50**_ **~ 0.1 μM) and a new cilostamide derivative MC2 (IC**_**50**_ **~ 1 μM).**

The test compound, MC2, was synthesized based on cilostamide structure by Department of Organic Chemistry, Mashhad University of Medical Sciences (Mashhad, Iran) according to the procedure reported by Sadeghian *et al*. and its PDE3 inhibitory action was assessed by Bioscience Company (BPS Bioscience Inc, San Diego, United States) using PDE assay Kit as described in previous works [14,20]. Figure 1 shows the IC50 of cilostamide, amrinone, and MC2 for PDE3. All the phosphodiesterase inhibitors were dissolved in dimethyl sulfoxide (DMSO) at a final concentration of 0.1% in the medium and equal amounts of carrier were added to control groups of the cells.

### Animals

Adult male Wistar rats with weights of 280–300 g were obtained from Laboratory Animal House, Mashhad University of Medical School, Iran. They were housed under a 12/12 h light/dark daily cycle at 22°C and had free access to standard foods and water. All experiments were conducted in accordance with standard ethical guidelines and approved by the local ethics committee of Mashhad University of Medical Sciences.

### Preadipocyte preparation and culture

The animals were sacrificed under ether anesthesia and retroperitoneal fat pads were removed immediately and placed in phosphate-buffered saline (PBS) supplemented with 100 U/ml penicillin and 100 μg/ml streptomycin in sterile condition. The tissue was minced into small pieces and incubated for 40 min in PBS containing 2 mg/ml collagenase at 37°C with mild agitation [21,22]. After centrifuging, the floated adipocytes were discarded and the stromal cells were suspended in DMEM medium supplemented with 10% fetal bovine serum, 100 U/ml penicillin and 100 μg/ml streptomycin and cultured in flask. After passage 3, the cells were seeded (at a density of 60000 cells/cm^2^) in 12-well plates. On next day, adipogenesis was induced by applying “induction medium” containing DMEM supplemented with 200 nM insulin, 250 μM IBMX, 1 μM dexamethasone, 200 μM indomethacin and 3% FBS. On day 3, the cells were subsequently cultured in “maintenance medium” (induction medium without IBMX and indomethacin) for 9 days and the medium was changed every 3 days.

To investigate the effect of PDE inhibitors on adipogenesis, IBMX was replaced with the same concentration of amrinone, cilostamide, or MC2 in the induction medium during the first three days of incubation. The cellular triglyceride accumulation was measured as an index of the adipocyte differentiation in the presence of PDE3 inhibitors and compared with IBMX.

### Oil Red O staining

Oil Red O was used to stain intracellular triglyceride droplets in differentiated adipocytes. Briefly, the cells were washed twice with PBS and fixed with 10% formalin for 30 min. After an additional washing with PBS, the cells were incubated with Oil Red O solution for 20 min [21,23]. Excess stain was removed by washing with distilled water and the stained cells were photographed. Lipid and Oil Red O were extracted using isopropyl alcohol and absorbance was measured using a spectrophotometer at a wavelength of 545 nm. The lipid content for each experimental group is expressed relative to that of IBMX-differentiated cells (adjusted to 100%).

### Determination of triglyceride

To support Oil Red O staining, the level of intracellular triglyceride (TG) was evaluated in differentiated adipocytes. Following washing the cells with PBS, intracellular TG was dissolved in 200 μl of 1% Triton X-100/PBS solution. Then, the level of TG was determined by TG assay kit (Pars azma, Co., Ltd., Iran). The protein concentrations were also measured by Bio-Rad protein assay dye reagent (Bio-Rad Laboratories, Inc.). Then the level of TG was normalized to cellular protein content of each treatment group [24]. The TG/protein content (mg/mg) was expressed as percentage compared to IBMX treated group as a control.

### Cell proliferation assay

To investigate the effects of the PDE inhibitors on adipocyte proliferation, preadipocytes were seeded (10^4^ cell/well) in flat-bottomed 96-well culture plates. After differentiation, adipocytes were incubated with amrinone, cilostamide and MC2 at the various concentrations (10, 100 and 500 μM) for 6, 12 and 24 h. After completion of the treatment, the cells were incubated with MTT solution for 3 h at 37°C. The supernatants were aspirated, DMSO was added to each well, and the plates were agitated to dissolve the crystal product [25-27]. Absorbance was measured at 545 nm (630 nm as a reference) using a StatFAX303 plate reader.

### Annexin V/PI double staining analysis

To detect the apoptosis induced by PDE inhibitors, Annexin V/PI double staining and flow cytometry analysis were used [28]. After adipocyte differentiation in 12-well plates, the cells were exposed to 10, 100 and 500 μM of MC2 for 24 h. After treatment, the cells were washed twice with cold PBS and resuspended in 100 μl binding buffer at a concentration of 1 × 10^6^ cells/ml. Then, 5 μl Annexin V-FITC and 10 μl PI (1 mg/ml) were added to these cells according to manufacturer’s instructions. Finally, the cells were analyzed with a FACScalibur flow cytometer (Becton Dickinson) and the distribution of normal, apoptotic and necrotic cells was calculated using WinMDI 2.7 software.

### Lipolysis assay

The differentiated adipocytes (cultured in 12-well culture plates) were pre-incubated with serum-free DMEM for 3 h and then bathed with 1 ml Krebs-Ringer bicarbonate buffer containing 5.5 mM glucose, 25 mM HEPES and 2% (w/v) bovine serum albumin. The cells were left untreated (basal lipolysis) or treated with isoproterenol (stimulated lipolysis) and incubated in the absence or presence of PDE inhibitors at 37°C in a humidified chamber under constant shaking for 2 h. Glycerol release in the medium was measured as index of lipolysis using the free glycerol determination kit [29,30].

### Statistical analysis

The results are presented as the mean ± standard error. The values were compared using the one-way analysis of variances followed by Dunnett’s post hoc test. The results were considered to be statistically significant, if the p-value was less than 0.05.

## Results

### Effect of PDE inhibitors on adipogenesis

**Figure 2:**
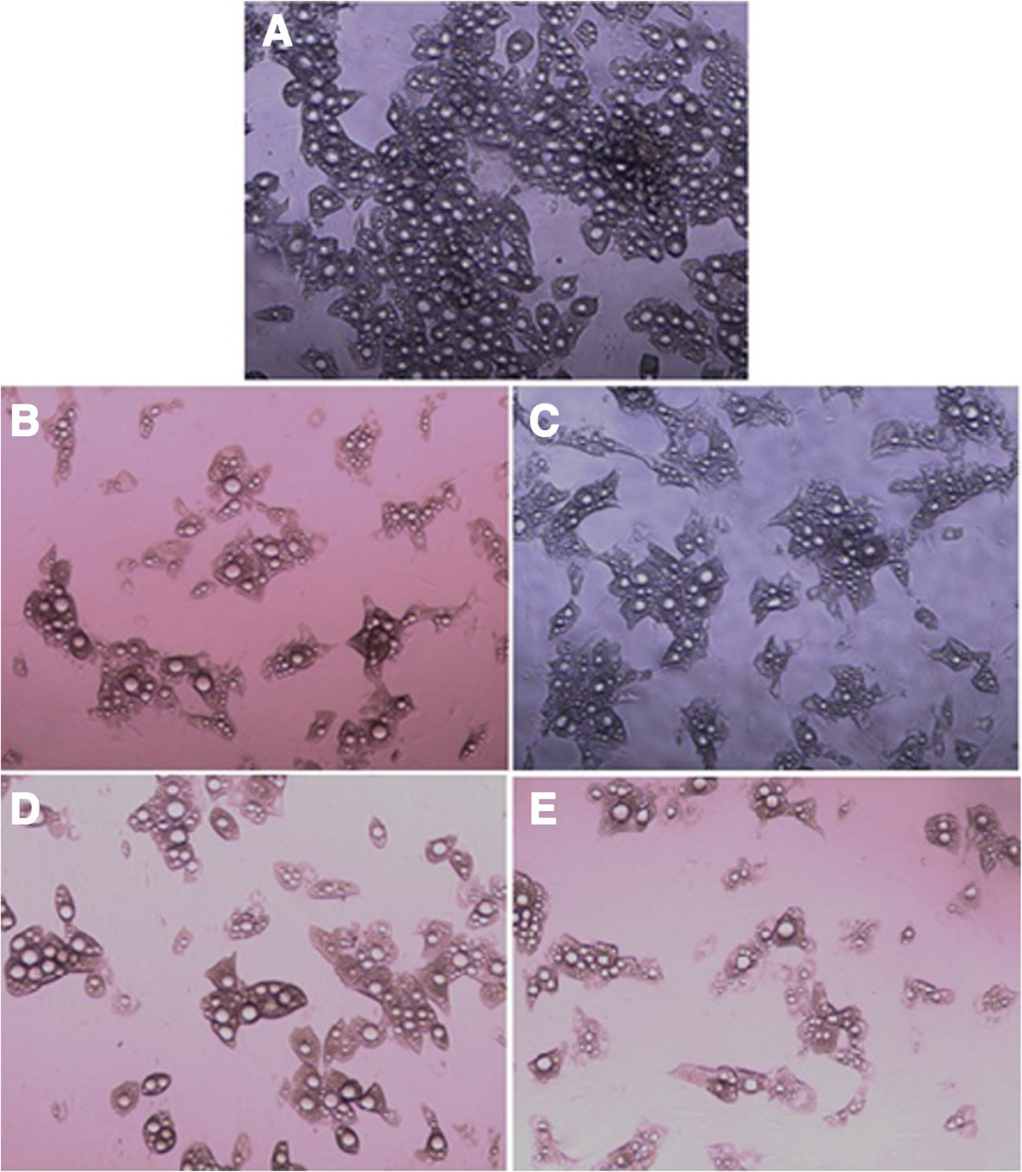
**Differentiation of preadipocyte to adipocyte in the presence of (A) IBMX, (B) no PDE inhibitor, (C) amrinone, (D) cilostamide and (E) MC2.** Adipogenesis was induced by 3 days incubation in “induction medium”; DMEM supplemented with 3% fetal bovine serum, 200 nM insulin, 250 μM of mentioned PDE inhibitors, 1 μM dexamethasone, 200 μM indomethacin. Then, the cells were subsequently cultured for 6 days in “maintenance medium” (induction medium without phosphodiestrase inhibitors and indomethacin). Magnification: ×100

The absence of IBMX in culture medium significantly decreased preadipocyte differentiation to adipocyte comparing to the control medium containing IBMX. Also, the level of adipogenesis in cilostamide and MC2 containing medium was lower than that of control medium. However, the effect of amrinone on preadipocyte differentiation was more than cilostamide and MC2, close to that of IBMX (Figure 2).

**Figure 3:**
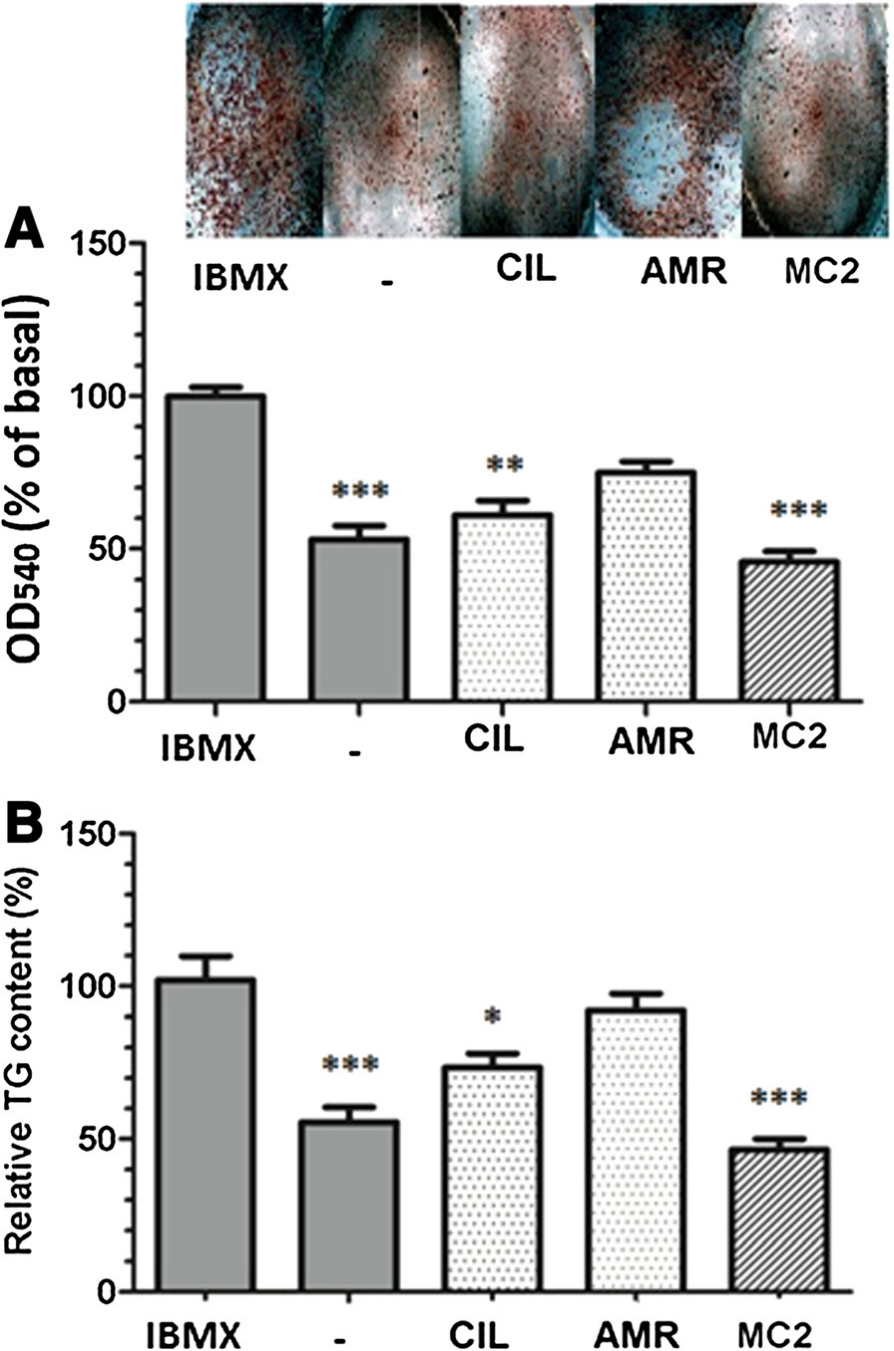
**Effects of phosphodiestrase inhibitors on adipogenesis.** Differentiation of preadipocytes was induced by 3 days incubation in induction medium; DMEM supplemented with 3% fetal bovine serum, 200 nM insulin, 1 μM dexamethasone, 200 μM indomethacin and 250 μM phosphodiestrase inhibitors (IBMX, amrinone (AMR), cilostamide (CIL), and (MC2 ). Then, the cells were subsequently cultured for 6 days in “maintenance medium” (induction medium without PDE inhibitors and indomethacin). Lipid accumulation was estimated by measuring the optical density (OD) of Oil Red O stain **(A)** or the level of triglyceride (TG) eluted from adipocytes **(B)**. Data are mean ± SEM of three independent experiments performed in triplicate. *p < 0.05,**p < 0.01 and ***p < 0.001 *vs* control (IBMX).

Oil Red O staining showed that in the presence of cilostamide and MC2 the level of lipid droplet accumulation was 69 ± 6% and 46 ± 4%, respectively, which was significantly lower than control medium containing IBMX (100 ± 4%, p < 0.01-p < 0.001) (Figure 3A). These findings were confirmed by measurement of intracellular TG in the differentiated adipocytes. As shown in Figure 3B, the level of intracellular TG in the presence of cilostamide (78 ± 6, P < 0.05) and MC2 (43 ± 5, P < 0.001) was lower than that of IBMX treated cells (100 ± 10).

### Effect of PDE inhibitors on adipocyte proliferation

**Figure 4:**
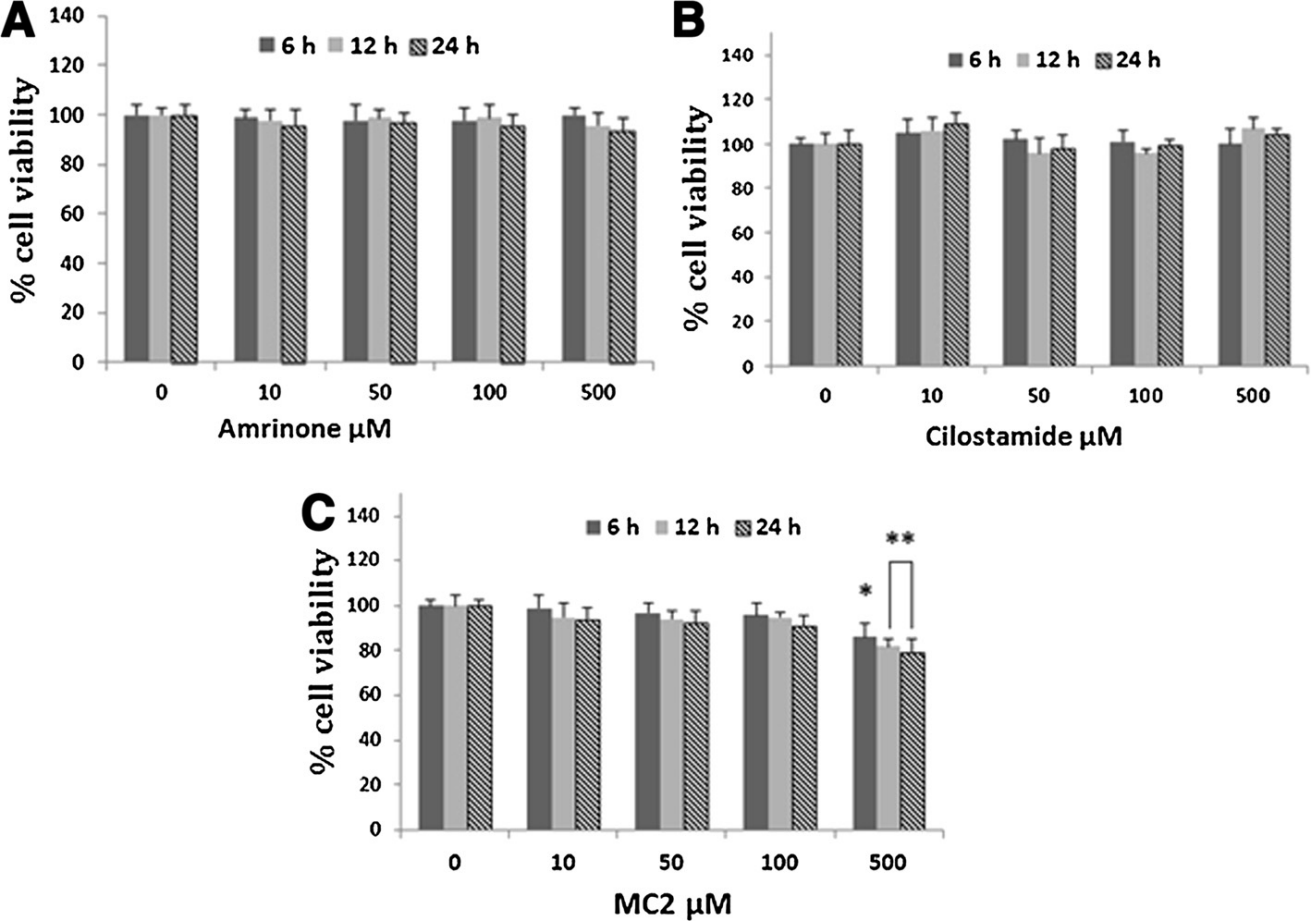
**Effects of phosphodiestrase inhibitors on viability of differentiated adipocytes.** The cells were incubated with various concentrations of amrinone **(A)**, cilostamide **(B)** and MC2 **(C)** for 6, 12 or 24 h. Cell viability was detected using MTT colorimetric assay. Values are mean ± SEM (n = 9). *p < 0.05 and **p < 0.01 *vs* control cells (0 μM).

Incubation of differentiated adipocytes with 10–500 μM of cilostamide, 10–500 μM of amrinoe and 10–100 μM of MC2 had no effect on their proliferation after 6, 12 and 24 h (Figure 4). MC2 at 500 μM significantly decreased proliferation of differentiated adipocyte to 86 ± 6 (p < 0.05), 82 ± 3 (p < 0.01) and 79 ± 6 (p < 0.01) after 6, 12 and 24 h, respectively.

### Effect of MC2 on adipocyte apoptosis

**Figure 5:**
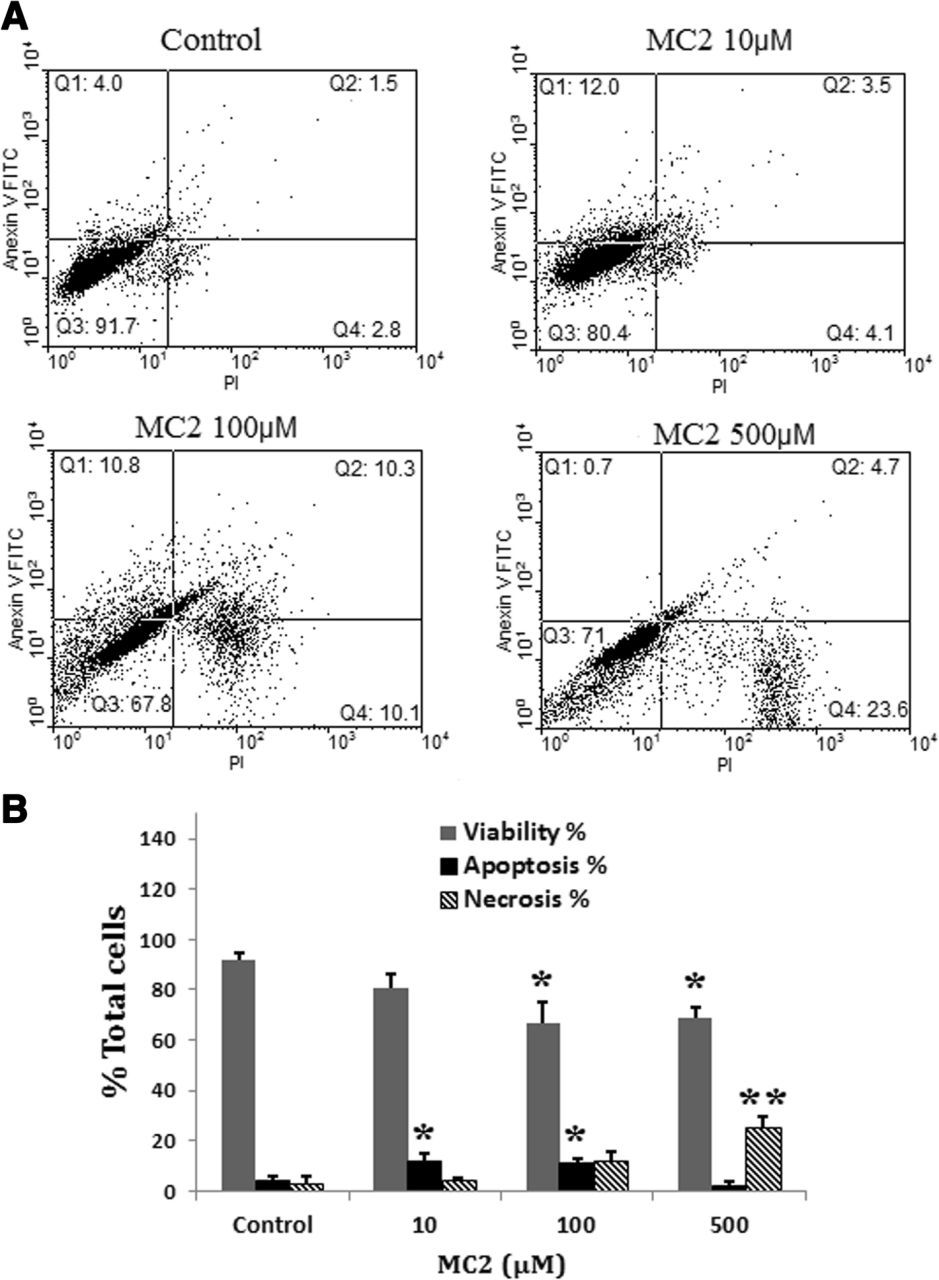
**Effect of MC2 on apoptosis of adipocyte. (A)** Detection of apoptosis and necrosis assessed with annexin-V-FITC and PI staining. The cells were treated with the indicated concentration of MC2 for 24 h. **(B)** Column bar graph of mean cell florescence for Annexin V-/PI- (Viable cells), Annexin V+/PI- (apoptotic cells), Annexin V+/PI+ (necrotic cells). Data are mean ± SEM of three experiments. *p < 0.05 and **p < 0.01 *vs* control.

Figure 5A shows the results of bivariate Annexin V/PI flow cytometry of differentiated adipocyte after 24 h incubation with MC2. The lower left quadrant of the histograms shows the viable cells, which exclude PI and are negative for FITC-Annexin V binding. The upper right quadrant represents the early apoptotic cells, which are PI negative and Annexin V positive, indicating integrity of the cytoplasmic membrane. The lower right quadrant represents the non-viable necrotic and late-stage apoptotic cells, which are positive for Annexin V binding and PI uptake. As shown in Figure 5B, incubation of differentiated adipocytes with MC2 caused the loss of cell viability at concentrations of 100 μM (p < 0.05) and 500 μM (p < 0.05). This effect was associated with the elevation in the number of apoptotic (concentrations of 10 and 100 μM, p < 0.05) and necrotic (concentrations of 500 μM, p < 0.01) cells.

### Effect of PDE inhibitors on lipolysis

**Figure 6:**
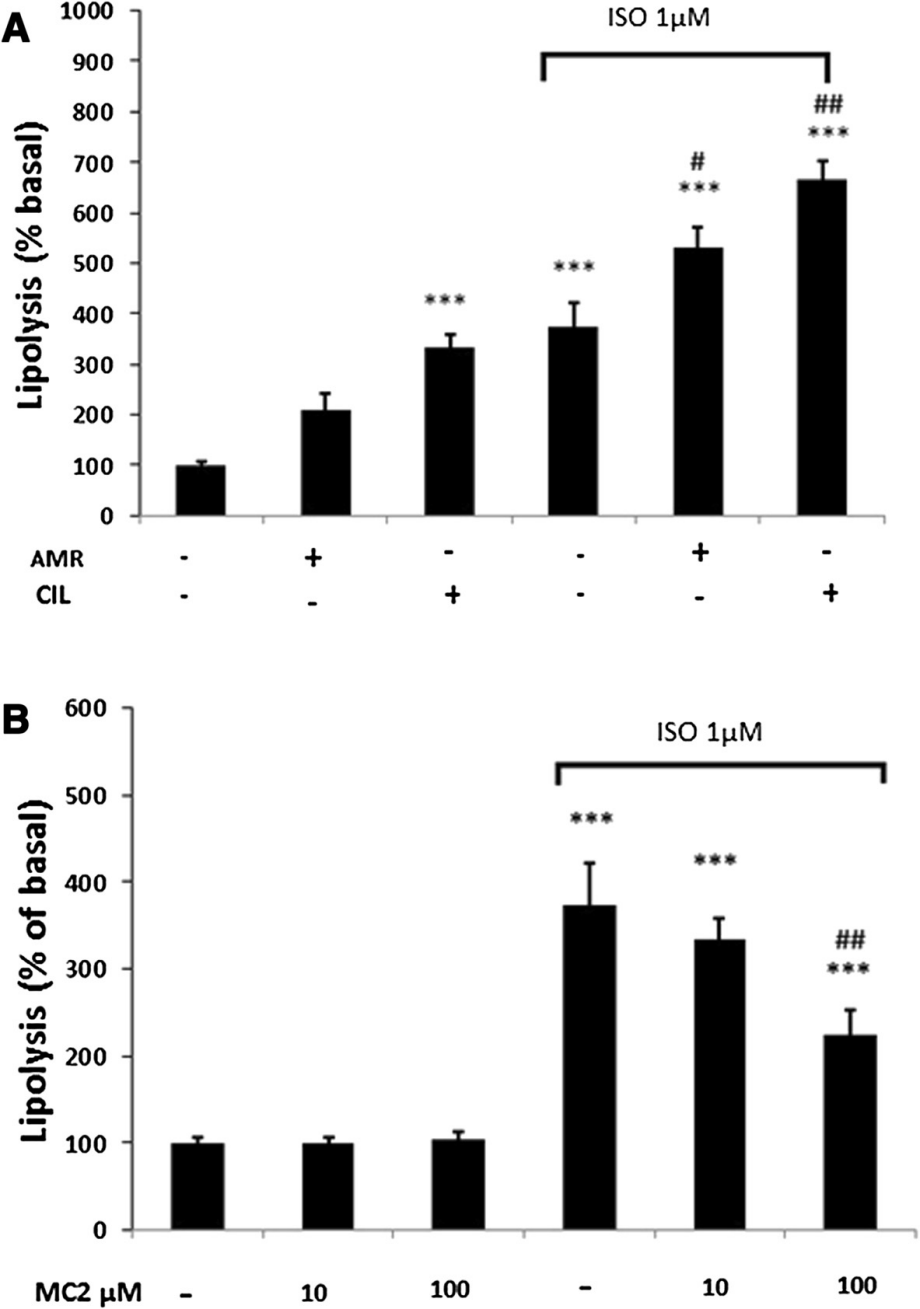
**Effects of phosphodiestrase inhibitors on basal and isoproterenol (ISO)-induced lipolysis.** Differentiated adipocytes were incubated with 100 μM amrinone (AMR), 100 μM cilostamide (CIL) **(A)** or indicated concentration of MC2 **(B)** for 120 min. Glycerol release in the culture media was assayed as lipolysis indicator. Data are mean ± SEM of three experiments. *P < 0.05 and ***P < 0.001 *vs* Control; #p < 0.05 and ##p < 0.01 vs ISO treated cells.

The differentiated adipocytes were incubated with different concentrations of PDE inhibitors and glycerol release was measured as index of lipolysis. Amrinone at 100 μM significantly increased isoproterenol-induced (but not basal) lipolysis (p < 0.05). Cilostamide at concentration of 100 μM significantly increased basal lipolysis from 100 ± 7% to 334 ± 24% (p < 0.001) and also enhanced stimulated lipolysis from 375 ± 48% to 668 ± 34% (p < 0.01) (Figure 6A). At the concentration of 10 and 100 μM, basal lipolysis was not changed by MC2. However, it significantly decreased isoproterenol-induced lipolysis from 374 ± 48 to 225 ± 27% (p < 0.01) at 100 μM (Figure 6B).

## Discussion

The PDEs play an important role in endocrine and cardiovascular functions, cell proliferation, cell differentiation, inflammation, and oxidative stress. Therapeutic application of PDE inhibitors, therefore, ranges from heart failure to pulmonary diseases to erectile dysfunction [31,32]. However, clinical application of these agents is limited because of their side effects, such as arrhythmia, impaired insulin secretion, and alterations in lipid metabolism [4,7]. Therefore, synthesis of new PDE3 inhibitors with desired pharmacological properties and lesser side effects is of great interest. In our previous work, we synthesized MC2 as a new cilostamide derivative and showed that it has inotropic action without unwanted effect on contraction rate [11,13]. In the present work, to assess the effect of synthesized compound on lipid metabolism, we investigated its effects on adipose tissue functions. Our data showed that MC2 unlike other PDE3 inhibitors does not increase adipogenesis and lipolysis and even has antilipolytic effect.

In agreement with previous reports, in our study the PDE3 inhibitors amrinone and cilostamide potentiated both basal and catecholamine (isoproterenol)-stimulated glycerol release from adipocyte [33]. However, MC2 not only did not increase lipolysis, but inhibited catecholamine-induced glycerol release. Previously, in an *in-vitro* study we showed that MC2 increases liver glycogen storage in rat and mouse which was different from those of other PDE inhibitors [12]. These effects of MC2 cannot be explained with its PDE3 inhibitory action because PDE inhibitors enhance lipolysis via elevation of intracellular cAMP [2].

In our previous studies we found that different PDE3 inhibitors produce differential cardiac and metabolic effects. All of test compounds produced positive inotropic effect but with different efficacies, which were not correlated with their IC_50_ of PDE3 inhibition. Also, they modified atrial contraction rates differently and some of which produced a negative chronotropic effect in spite of having a high positive inotropic effects [11]. Also, in rat hyperglycemic clamp model MC2 increased insulin secretion similar to the other tested PDE inhibitors, but opposite to others it increased the level of glycogen in the liver [12]. These effects implicate that a cAMP-independent signaling pathways may be involved in the biological effects of PDE inhibitors which is manifested by MC2 and may mediate its antilipolytic effect.

In patients with poorly controlled diabetes, deficiency of insulin in conjunction with catecholamine- and glucagon-stimulated lipolysis enhances fatty acid delivery to liver which may lead to ketoacidosis and even death [19]. It is reasonable to conclude that MC2 can decrease the risk of ketoacidosis through inhibition of lipolysis in diabetic patients who are candidate to PDE inhibitor therapy due to cardiovascular diseases.

Consistent with previous reports on the role of PDE inhibitors in adipogenesis [16,34], we found that deprivation of differentiation medium from IBMX attenuated adipogenesis. Neither cilostamide nor MC2 could restore the level of adipocyte differentiation to that of IBMX treated group. It has been revealed that PDEs exist in two forms: soluble (cytosolic) and particulate (membrane-associated) [35]. It is generally accepted that inhibition of soluble but not particulate PDE activity is responsible for IBMX-stimulated differentiation of pre-adipocytes [36]. Therefore, inhibition of PDE3 which predominately exist in the form of particulate cannot mimic the role of IBMX in adipocyte differentiation. On the other hand, similar to what happens in the heart and kidney [37], amrinone, in high concentration may inhibit soluble PDE and therefore could induce adipocyte differentiation approximately to the level induced by IBMX.

The mass of adipose tissue is determined by size and number of adipocytes. The size of adipocytes is reduced by lipolysis and increased by lipogenesis [38,39]. Number of adipocytes is directly related to the rate of adipogenesis and inversely related to the rate of apoptosis. Results of Annexin V/PI and MTT assays showed that MC2 increases adipocyte apoptosis. However, this effect was produced at high concentrations which reduced cell viability and proliferation. Since MC2 cannot increase adipogenesis, its potential proapoptotic effect may reduce total adipocytes number in fat tissue. However, antilipolytic effect of MC2 may prevent severe alteration in the mass of adipose tissue. Future works are needed to address long term effect of MC2 on fat mass.

In conclusion, our data showed that different types of PDE3 inhibitors induce different effects on lipolysis and adipogenesis. Here we introduced MC2 as a new PDE3 inhibitor with potential proapoptotic and antilipolytic effects on adipocytes and without stimulatory action on adipogenesis. These effects of MC2 implicate that, in comparison with amrinone or milrinone, this drug may have lowest metabolic side effect and might be a good candidate for treatment of congestive heart failure.
